# Intracorporeal versus extracorporeal anastomosis in segmental resections for colon cancer: a retrospective cohort study of 328 patients

**DOI:** 10.1007/s00423-023-02946-w

**Published:** 2023-05-31

**Authors:** Pedja Cuk, Musa Büyükuslu, Sören Möller, Victor Jilbert Verwaal, Issam Al-Najami, Mark Bremholm Ellebæk

**Affiliations:** 1 Department of General and Colorectal Surgery, University Hospital of Southern Denmark, Kresten Philipsens Vej 15, 6200 Aabenraa, Denmark; 2 Institute of Regional Health Research, University of Southern Denmark, Pedja Cuk, Billesgade 14, 5000 Odense C, Denmark; 3 Surgical Department, Hospital of South West Jutland, Finsensgade 35, 6700 Esbjerg, Denmark; 4 Department of Clinical Research, University of Southern Denmark, J.B. Winsloewsvej 19, 5000 Odense, Denmark; 5 Clinical Research Center, Lund University, Jan Wäldenströms Gata 35, 20502 Malmö, Sweden; 6 Research Unit for Surgery, Odense University Hospital and University of Southern Denmark, J.B. Winsloewsvej 4, 5000 Odense, Denmark

**Keywords:** Intracorporeal anastomosis, Extracorporeal anastomosis, Anastomosis technique, Minimally invasive surgery, Colon cancer

## Abstract

**Purpose:**

The intracorporeal anastomosis (IA) technique possibly results in enhanced recovery and reduced morbidity rates compared to the extracorporeal anastomosis (EA) technique. This study compared the short-term morbidity rates of IA versus EA in segmental resections for colon cancer.

**Method:**

We performed a retrospective cohort study of consecutive patients from 2015 to 2020 using the IA or EA technique at a single Danish colorectal center. Comparative outcomes of interest were surgical efficacy and short-term morbidity rates. An inverse probability of treatment weighting (IPTW) analysis of clinically relevant outcomes was conducted to explore potential baseline confounding.

**Results:**

We included 328 patients, 129 in the EA and 199 in the IA groups. There was no significant difference in preoperative baseline characteristics between the two groups. The rate of overall surgical (16% in both groups, *p* = 1.000) and medical complications (EA: 25 (19%) vs. IA: 27 (14%), *p* = 0.167) was comparable for both groups. The IA technique did not cause a reduction in operative time (EA: 127.0 min [103.0–171.0] vs. IA: 134.0 min [110.0–164.0], *p* = 0.547). The IPTW analysis indicated that having an IA caused a reduction in the rate of major surgical complications (RRR_adjusted_ = 0.45, 95%CI [0.29–0.69], *p* = 0.000).

**Conclusion:**

Adopting IA for colon cancer resulted in similar overall morbidity rates without increasing the duration of the surgical procedure compared to EA. The IA technique had a probable protective effect against developing severe surgical complications. However, this must be interpreted cautiously, limited by the retrospective study design.

## Introduction

Previous studies suggest an association between the IA and enhanced recovery rates, improved overall morbidity rates, and reduced surgical site infection rates compared to the EA technique [1–3]. These results are primarily reported in studies using the IA in right-sided colonic cancer surgery and are of a retrospective design [1–6]. However, the studies are currently limited by smaller sample sizes, including patients operated on for benign and malignant conditions in a planned and emergent setting. This fact may challenge definite conclusions. On the contrary, randomized controlled studies, including smaller sample sizes in patients having right-sided colectomies with IA or EA, suggest that IA is associated with a faster time to bowel function and reduced postoperative pain. No benefits of IA were found concerning postoperative morbidity or recovery rates [7–9].

Few studies compared the potential pathophysiological differences between the two anastomotic techniques. It is believed that EA is associated with a more significant surgical trauma because of longer skin incisions needed to access the bowel manually. Performing the EA in an extra-abdominal environment may also contribute to greater traction to the mesentery, increased risk of serosal injuries, and intraoperative bleeding causing impaired postoperative recovery, prolonged intestinal paralysis, and higher morbidity rates [10].

This study aims to investigate the short-term morbidity, safety, and efficacy of IA versus EA in all segmental colonic resections for malignancy performed in a large tertiary colorectal referral center since implementing the IA technique.

## Methods

### Study design

This retrospective cohort study was reported using the Strengthening Reporting of Observational Studies in Epidemiology (STROBE) guidelines [11]. It was approved by the National Institutional Board, NO 2008–58-0035. Data on adult patients operated consecutively from June 2015 until June 2020 with either IA or EA since the implementation of the IA technique at the Surgical Department of the Hospital of South West Jutland were compared. All patients who underwent minimally invasive planned surgery for colon cancer were included in the study. Patients were excluded if they underwent colon resection in case of benign etiology, non-malignant pathology (polyps), emergency resections, stoma formation, and procedures combining both IA and EA. Data were retrospectively reviewed from medical chart records. Patients were consecutively identified from unique personal numbers and linked to respective treatment and procedure ICD-10 codes (KJFB00, 01, 10, 20, 21, 30, 30A, 30B, 31, 31A, 31B, 44, 47). Medical charts were reviewed independently and checked by a single author (MB). Demographic, intra-, and postoperative data were registered in a designated database. Demographic data included the following variables: gender, age, BMI, ASA score, existing comorbidity, WHO performance status, tobacco and alcohol consumption, history of previous abdominal surgery, tumor (T), and nodal (N) staging. Data regarding intraoperative details included intraoperative conversion from EA to IA, surgical time consumption, time to mobilization, time to first flatus and stool, length of hospital stay, and estimated blood loss. Postoperatively, we recorded 30-day morbidity rates, including surgical and medical complications according to the Clavien-Dindo classification [12], 30-day mortality rates, and comprehensive complication index (CCI) [13]. Anastomotic leakage was rated into three severity grades (A, B, or C) and was defined as an intestinal disruption diagnosed by either computed tomography, surgery, or endoscopy [14].

### Surgical procedure

According to the Danish national consensus, a specialist in oncological colorectal surgery was required to participate in the procedure. Patients with suspected colon cancer underwent an oncological D2-resection. In case an EA was performed, a right-sided horizontal, a left-sided subcostal median, or a Pfannenstiel incision was applied depending of the type of resection and surgeon’s preference. In the IA group, the total surgical procedure was accomplished without exteriorizing the bowel, and the intestinal ends were transected with a linear stapler. An enterotomy was performed by incising the bowel, and a side-to-side isoperistaltic anastomosis was performed using Endo GIA® gold tri-stapler, Medtronic, USA. The enterotomy was closed with a continuously running multifilament absorbable suture in two layers. The mesentery defect was left open, and the specimen was extracted through a Pfannenstiel incision. Patients, where an intended IA was not performed due to technical difficulties, were converted to an EA perioperatively. Antibiotics were preoperatively administered by routine, while postoperative administration depended on the surgeon’s choice.

### Statistics

In the univariate analysis, categorical data were presented by relative frequencies and percentages and compared using Fischer’s exact test. Continuous variables were presented in the median and interquartile range (IQR), and the groups were compared with a Wilcoxon rank-sum test under the assumption of non-normal distribution. We decided to perform an IPTW analysis to minimize the risk of selection bias and baseline confounding interaction using propensity scores due to the heterogeneous patient population included in our study [15]. After balancing the population, multivariate logistic regression was conducted. Baseline confounding was examined using the following covariates: age, gender, BMI, ASA score, T-stage, type of surgical platform (laparoscopic or robot-assisted), surgical procedure (right- or left-sided resection), and history of previous abdominal surgery. We explored outcomes of interest from the multivariate analyses and included the CCI, overall surgical and medical complication rate, length of hospital stay, and the estimated blood loss. All analyses were performed in STATA (StataCorp. 2017. Stata Statistical Software: Release 15. College Station, TX: StataCorp LLC). A *p* value of < 0.05 was considered statistically significant.

## Results

**Fig. 1:**
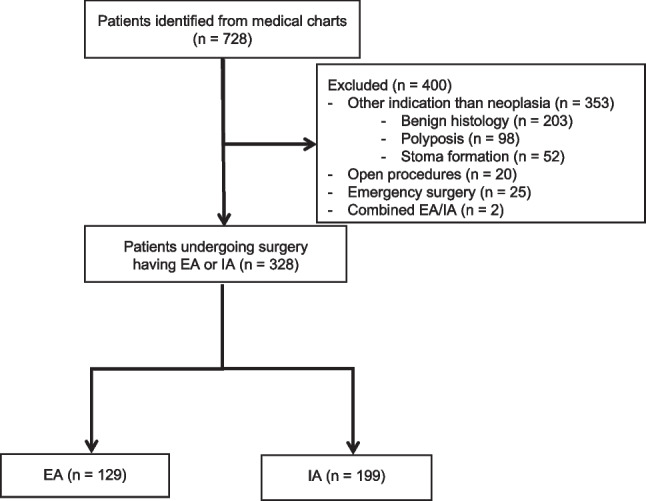
Study flowchart. EA = extracorporeal anastomosis, IA = intracorporeal anastomosis

From June 2015 until June 2020, 728 patients were identified from medical chart records. After the exclusion of 400 patients, 328 patients (199 in the IA and 129 in the EA group) were included in the final analysis (Fig. 1). No significant differences between the two groups were observed in baseline characteristics, including the patient’s preoperative morbidity index, ASA score, and BMI (Table 1). According to Table 2, a higher proportion of patients undergoing left-sided resections had an EA, while patients having right-sided resections were overrepresented in the IA group.

**Table 1:** Baseline characteristics of patients included in the study

Outcome	EA (*n* = 129)	IA (*n* = 199)	*p*
Sex			
Male (*n*, %)	66 (51%)	117 (59%)	0.211
Female (*n*, %)	63 (49%)	82 (41%)	
Age (median/IQR)	72.0 (66.0–77.0)	73.0 (68.0–79.0)	0.102
BMI (median/IQR)	26.1 (23.8–29.6)	25.8 (22.9–29.3)	0.119
ASA (1/2/3/4, *n*, %)	11/73/43/2 9%/57%/33%/2%	8/119/67/4 4%/60%/34%/2%	0.392
Comorbidity			
Cardiac (*n*, %)	27 (21%)	50 (25%)	0.425
Pulmonary (*n*, %)	19 (15%)	38 (19%)	0.371
Renal (*n*, %)	8 (6%)	10 (5%)	0.631
Hypertension (*n*, %)	65 (50%)	103 (52%)	0.821
Diabetes mellitus (*n*, %)	18 (14%)	26 (13%)	0.869
WHO performance status (0/1/2/3/4, *n*, %)	61/16/6/0/1 73%/19%/7%/0%/1%	67/35/13/2/0 57%/30%/11%/2%/0%	0.078
Smoking (*n*, %)	18 (15%)	32 (16%)	0.604
Alcohol consumption			
< 14 units/week	85 (68%)	136 (70%)	0.937
> 14 units/week	6 (5%)	10 (5%)	
Prior abdominal surgery (*n*, %)	51 (40%)	66 (33%)	0.241
T-stage (1/2/3/4)	10/17/78/18 (8%/14%/63%/15%)	17/31/111/32 (9%/16%/58%/17%)	0.839
N-stage (0/1/2)	74/34/15 (60%/28%/12%)	113/60/22 (58%/31%/11%)	0.851

**Table 2:** Intra- and postoperative data regarding surgical efficacy. *EA =* extracorporeal, *IA* = intracorporeal. Bold values indicates statistical significance at the *p* < 0.05 level

Outcome	EA (*n* = 129)	IA (*n* = 199)	*p*
Surgical procedures			
Right-sided hemicolectomy (*n*, %)	12 (9%)	82 (41%)	**0.000**
Extended right-sided hemicolectomy (*n*, %)	9 (7%)	87 (44%)	
Left-sided hemicolectomy (*n*, %)	14 (11%)	29 (15%)	
Sigmoid resection (*n*, %)	94 (73%)	1 (1%)	
Operative duration, min (median [range])	127.0 (103.0–171.0)	134.0 (110.0–164.0)	0.547
Estimated blood loss, mL (median [range])	50.0 (40.0–100.0)	50.0 (50.0–100.0)	0.099
Time to mobilization, days (median [range])	0.5 (0.3–0.9)	0.5 (0.3–0.8)	0.756
Time to flatus, days (median [range])	1.0 (0.9–1.9)	1.4 (1.0–2.0)	**0.003**
Time to stool, days (median [range])	2.0 (1.4–3.0)	2.3 (1.9–3.1)	**0.004**
Length of stay, days (median [range])	3.4 (2.9–5.0)	3.2 (2.9–5.2)	0.956

Perioperative data on surgical efficacy in the EA versus IA group demonstrated no statistical difference in operation time between the two groups (EA: 127.0 min (103.0–171.0), IA: 134.0 min (110.0–164.0), *p* = 0.547). A total of four patients in the IA group (2%) had the anastomosis converted perioperatively to EA due to technical difficulties. The EA, compared to the IA group, resulted in a faster time to bowel function (1.0 days (0.9–1.9) vs. 1.4 days (1.0–2.0), *p* = 0.003), while the length of stay did not differ statistically (EA = 3.4 days (2.9–5.0), IA = 3.2 days (2.9–5.2), *p* = 0.956) (Table 2).

In the postoperative course, patients with an EA had a higher amount of anastomotic leakage than IA (11% vs. 6%, *p* = 0.039). However, the rate of overall surgical complications (16% in both groups, *p* = 1.000) and overall medical complications (19% vs. 14%, *p* = 0.167) did not differ between the two groups (Table 3). The remaining parameters (CCI and 30-day mortality rate) did not reach any statistically significant difference between the two groups.

**Table 3:** Postoperative outcomes of short-term morbidity. *EA =* extracorporeal, *IA =* intracorporeal, *CCI* = comprehensive complication index. Bold values indicates statistical significance at the *p* <0.05 level

Outcome	EA (*n* = 129)	IA (*n* = 199)	*p*
Overall surgical complications (*n*, %)	20 (16%)	31 (16%)	1.000
Surgical complications (*n*, %)			
Hemorrhage	3 (2%)	11 (6%)	0.263
Bowel obstruction	8 (6%)	10 (5%)	0.631
Anastomotic leakage	11 (9%)	6 (3%)	**0.039**
Surgical wound infection	2 (2%)	2 (1%)	0.648
Clavien-Dindo I–II surgical complications (*n*, %)	4 (3%)	14 (7%)	0.200
Clavien-Dindo III–IV surgical complications (*n*, %)	16 (12%)	17 (9%)	0.200
Overall medical complications (*n*, %)	25 (19%)	27 (14%)	0.167
Medical complications (*n*, %)			
Pneumonia	5 (4%)	5 (3%)	0.523
Sepsis	3 (2%)	1 (1%)	0.304
Heart failure	2 (2%)	2 (1%)	0.648
Other	18 (14%)	19 (19%)	0.284
Anemia	2 (2%)	6 (3%)	0.488
Bacteremia	8 (6%)	10 (5%)	0.631
Diarrhea	3 (2%)	0 (0%)	0.060
Constipation	2 (2%)	0 (0%)	0.154
Delirium	2 (2%)	1 (1%)	0.564
Dehydration	1 (1%)	0 (0%)	0.393
Subcutaneous emphysema	0 (0%)	1 (1%)	1.000
Renal failure	0 (0%)	1 (1%)	1.000
Clavien-Dindo I–II medical complications (*n*, %)	21 (16%)	26 (13%)	0.215
Clavien-Dindo III–IV medical complications (*n*, %)	3 (2%)	1 (1%)	0.215
CCI (mean, SD)	8.6 (16.0)	7.2 (15.1)	0.447
30-day mortality, (*n*, %)	0 (0%)	2 (1%)	0.521

According to Table 4, the CCI favored IA in both the non-adjusted (MD = - 1.33, 95%CI [− 4.78, − 2.11], *p* = 0.447) and adjusted IPTW analyses (MD =  − 7.89, 95%CI [− 11.19, − 4.60], *p* = 0.000). Patients having IA had a higher odds of developing non-severe surgical complications (RRR_adjusted_ = 3.43, 95%CI [2.05–5.79], *p* = 0.000), while this anastomotic technique had a protective effect for developing major surgical complications (RRR_adjusted_ = 0.45, 95%CI [0.29–0.69], *p = 0.000*). The IA technique caused a reduction in non-severe medical complications (RRR_adjusted_ = 0.62, 95%CI [0.42–0.91], *p* = 0.014), while the benefit was less pronounced in the case of severe medical complications (RRR_adjusted_ = 0.03, 95%CI [0.00–2.07], *p* = 0.000).

**Table 4:** Inverse probability of treatment weighting (IPTW) analysis. Reference is considered as intracorporeal anastomosis group. *CCI* = comprehensive complication index, *CD* = Clavien-Dindo classification, *MD* = mean difference, *RRR* = relative risk reduction, *95%CI* = confidence interval. Bold values indicates statistical significance at the *p* < 0.05 level

Variables	Non-IPTW analysis			IPTW-adjusted analysis		
	MD/RRR	95%CI	*p*	MD/RRR	95%CI	*p*
CCI	− 1.33	− 4.78, − 2.11	0.447	− 7.89	− 11.19, − 4.60	**0.000**
Surgical complications, CD 1–2	2.27	0.72–7.08	0.157	3.43	2.05–5.79	**0.000**
Surgical complications, CD 3–4	0.69	0.33–1.42	0.314	0.45	0.29–0.69	**0.000**
Medical complications, CD 1–2	0.76	0.40–1.41	0.379	0.62	0.42–0.91	**0.014**
Medical complications, CD 3–4	0.20	0.02–1.98	0.170	0.03	0.00–2.07	**0.000**
Length of stay	− 0.71	− 1.98, 0.56	0.274	− 5.70	− 8.14, − 3.25	**0.000**
Estimated blood loss	− 13.29	− 46.83, − 20.24	0.436	8.08	− 7.14, − 23.30	0.298

## Discussion

In this study, we showed that adopting the IA technique for colon cancer resulted in comparable overall short-term morbidity rates without impairing the surgical efficacy compared to the EA technique. However, the IA technique was associated with a significant reduction in the rate of severe surgical complications, and it caused a shortened time of hospitalization (Table 4).

Since the first case series reports of minimally invasive colonic surgery in 1991 [16] were published, there has been an ongoing interest in minimizing surgical trauma. Due to the increased experience within laparoscopic colonic surgery, a greater interest has steadily increased in performing the majority of the surgical procedure intracorporeally. Several observational studies, RCTs, and meta-analyses have recently compared the EA versus IA in right-sided colonic resections of predominantly malignant etiology [2, 3, 5, 7–9, 17–22]. One of the main concerns when adopting the IA technique has been the risk of surgical site infections due to the manipulation of the open bowel intracorporeally when performing the anastomosis. However, studies have indicated an increased risk of surgical site infections using the EA technique, probably explained by an increased risk of fecal contamination [8, 21]. The surgical site infection rate was comparable between the two groups in our study. One of the main advantages when performing the IA is the ability to extract the resected specimen anywhere on the abdominal wall, thereby avoiding midline incisions associated with a higher incidence of incisional hernia [23]. Additional benefits of IA include minimized traction of the bowel mesentery preventing unnecessary trauma, bleeding, and serosal injuries. This factor can contribute to a reduced rate of paralytic ileus resulting in lower postoperative consumption of analgesics, less pain, a faster establishment of bowel function, and a shorter hospitalization time [20–22].

Prospective studies and RCTs cannot determine significant differences in postoperative complication rates, apart from a faster recovery time, discharge, and reduced postoperative pain between the two anastomotic techniques. However, this may be due to smaller sample sizes [7, 9]. Several systematic reviews and meta-analyses, including studies with a heterogeneous design, have demonstrated improved short-term morbidity, including decreased anastomotic leakage- and conversion rates, incisional hernia, and surgical site infections in favor of IA predominantly in right-sided colonic cancer [20–22]. It should be mentioned that the anastomotic leakage rate in our study was higher in the EA compared to the IA group in the univariate analyses, explained by a higher prevalence of patients with left-sided colon cancer in the EA group. However, according to the multivariate regression and IPTW analyses (Table 4), when balancing the two groups and adjusting for the type of resection (right- or left-sided), the IA group resulted in a significant reduction of severe surgical complications and CCI. In our series, a clinically relevant reduction in the length of hospital stay could be confirmed in the IA compared to the EA group (Table 4). The disadvantage of the IA technique is increased surgical time, as the laparoscopic intracorporeal suturing of the enterotomy is more complex and demanding to perform than the EA technique [8, 24]. This problem was not reflected in our results, and the time duration of surgery did not differ between the two groups in the univariate analyses despite including the implementation phase.

This study has several limitations due to its retrospective design leaving a high risk of unmeasured confounding. However, it should be emphasized that only experienced senior colorectal surgeons have operated on patients since implementing the IA technique; the data presented include all patients managed in the period using the IA technique, thus contributing to representable data completeness and transparency after introducing this anastomosis technique at the department. Additionally, we performed an IPTW analysis to strengthen the results of our univariate analyses. We also reduced the risk of baseline confounding and selection bias by equalizing the distribution of confounders and balancing the patient’s baseline characteristics [25]. Our short-term morbidity and recovery rates data can be considered reliable despite the retrospective study design and intergroup variance in the population included. In addition, the adjusted IPTW analysis demonstrated a reduction of medical complications (Clavien-Dindo grade 1–2). However, it is common that the Clavien-Dindo classification of complications may underestimate the amount of non-severe complications and their prognostic implication following colonic surgery [26].

Adopting the IA technique for colon cancer resulted in similar overall short-term morbidity rates without impairing the surgical efficacy compared to the EA. However, IA was associated with a reduced rate of severe surgical complications and CCI.

## Data Availability

Data available upon reasonable request from the authors.
